# Experience Drives Synchronization: The phase and Amplitude Dynamics of Neural Oscillations to Musical Chords Are Differentially Modulated by Musical Expertise

**DOI:** 10.1371/journal.pone.0134211

**Published:** 2015-08-20

**Authors:** Karen Johanne Pallesen, Christopher J. Bailey, Elvira Brattico, Albert Gjedde, J. Matias Palva, Satu Palva

**Affiliations:** 1 Department of Neuroscience and Pharmacology, University of Copenhagen, Copenhagen, Denmark; 2 The Research Clinic for Functional Disorders and Psychosomatics, Aarhus University Hospital, Aarhus, Denmark; 3 Center of Functionally Integrative Neuroscience, Aarhus University, Aarhus, Denmark; 4 Helsinki Collegium for Advanced Studies, University of Helsinki, Helsinki, Finland; 5 Cognitive Brain Research Unit, Institute of Behavioral Science, University of Helsinki, Helsinki, Finland; 6 Pathophysiology and Experimental Tomography Center, Aarhus University Hospital, Aarhus, Denmark; 7 Neuroscience Center, University of Helsinki, Helsinki, Finland; 8 BioMag laboratory, HUS Medical Imaging Center, Helsinki University Central Hospital, Helsinki, Finland; ARC Centre of Excellence in Cognition and its Disorders (CCD), AUSTRALIA

## Abstract

Musical expertise is associated with structural and functional changes in the brain that underlie facilitated auditory perception. We investigated whether the phase locking (PL) and amplitude modulations (AM) of neuronal oscillations in response to musical chords are correlated with musical expertise and whether they reflect the prototypicality of chords in Western tonal music. To this aim, we recorded magnetoencephalography (MEG) while musicians and non-musicians were presented with common prototypical major and minor chords, and with uncommon, non-prototypical dissonant and mistuned chords, while watching a silenced movie. We then analyzed the PL and AM of ongoing oscillations in the theta (4–8 Hz) alpha (8–14 Hz), beta- (14–30 Hz) and gamma- (30–80 Hz) bands to these chords. We found that musical expertise was associated with strengthened PL of ongoing oscillations to chords over a wide frequency range during the first 300 ms from stimulus onset, as opposed to increased alpha-band AM to chords over temporal MEG channels. In musicians, the gamma-band PL was strongest to non-prototypical compared to other chords, while in non-musicians PL was strongest to minor chords. In both musicians and non-musicians the long-latency (> 200 ms) gamma-band PL was also sensitive to chord identity, and particularly to the amplitude modulations (beats) of the dissonant chord. These findings suggest that musical expertise modulates oscillation PL to musical chords and that the strength of these modulations is dependent on chord prototypicality.

## Introduction

Musical training leads to expertise in the auditory domain, evidenced as behavioral advantages in perceptual skills related to sound features, as well as to non-musical sounds [1]. These include perceptual acuity for small sound differences in music [2, 3], voice speech cues, pitch contour in speech (prosody), phonetic advantage for second language, speech-in-noise perception, cognitive control in auditory working memory [4], temporal synchronization [5], and the advancement of other cross-domain functions [1, 6]. The sound-related superiority in music experts depends on experience-dependent plasticity, namely structural and functional changes in the brain that are behaviorally relevant in musical tasks [1, 7–9].

Music-derived neuroplasticity of the auditory cortex can be investigated non-invasively with electroencephalography (EEG) and magnetoencephalography (MEG) recordings by comparing sound-induced neuronal activity between musicians and non-musicians. Several studies show that musical expertise leads to an enhanced recruitment of neuronal resources to process (musical or non-musical) sounds, and particularly salient ones, i.e., the sounds that have a particular significance to individuals due to repeated exposure and ‘sound to meaning’ associations [1]. The facilitated neuronal processing of such salient and subjectively relevant sounds in musicians are reflected in the increased amplitudes of averaged enhanced middle-latency (19–30 ms) event-related responses (ERs, also called event-related potentials or ERPs) to pure tones [10], and enhanced later event-related ERs [11–17]. EEG [10] and MEG [13] studies show that both N1 and P2 responses are enhanced in musicians during passive listening to tones. Furthermore, the pre-attentively elicited mismatch negativity (MMN) response (~100–200 ms) to violations of repetitive (standard) features in a musical sequence (e.g. a new pitch in a succession of repeated tones) is enhanced in musicians compared to non-musicians [16]. The MMN to sound feature changes is generally assumed to rely on the ongoing formation of a sensory memory template or prediction for sound features based on the statistical properties of the sound environment, and on the feed-forward matching of this template to the incoming sounds [18].

Acoustic training in the laboratory lasting from minutes to several weeks in non-musicians yields similar effects to long-term musical training, enhancing the strength of N1 and P2 ERs [11, 19–21]. For example, frequency change detection with 40-Hz amplitude modulated pure tones improved after 15 sessions and led to enhanced P2 bilaterally and N1c in the right hemisphere [11] and as little as an hour of discrimination training with pure tones was found to modulate the N1 response [19]. Auditory temporal sensitivity also improves after just three hours of training, reflected in enhanced ER amplitudes [21]. These results demonstrate that training-induced plasticity is the likely cause of differences in neuronal activity between musicians and non-musicians, as reflected by the amplitude of the neural currents or magnetic fields recorded from the scalp.

In addition to ERs, stimulus processing is reflected in the dynamics of cortical oscillations. Oscillations and synchronized neural activity regulate and coordinate processing of visual percepts [22–24], and specifically gamma oscillations have been suggested to underlie the integration of visual features [25].

In humans, sensory stimuli evoke early gamma oscillations, which are sensitive to physical stimulus features both in the auditory [26, 27] and visual modalities [28] as well as later non-phase-locked induced oscillations of which amplitude modulations reflect the neural processing of higher level perceptual features of visual stimuli [29–36]. In the auditory modality, observations of stimulus-specific induced gamma oscillations are less frequent and have been observed in perceptual tasks [36, 43–45] and in auditory working memory tasks [27, 37–40].

Oscillations and synchronized neuronal activity play a crucial role in coordinating activity-dependent synaptic plasticity [41, 42]. Hence, oscillations are a likely target for experience-induced plasticity modulations by musical experience. Indeed, early evoked gamma band oscillations have been suggested to underlie feature binding of well-learned memory traces [21], and matching of feed-forward activation to a memory template [26, 43] and expectations [44]. Therefore both amplitude and phase-dynamics of gamma-band oscillations might reflect plastic modulations in the perception of sensory representations. In the music domain, it has been found that the familiarity with a musical timbre enhances both the evoked and induced gamma band responses to individual tones, compared to a less familiar timbre [45]. Further, evoked gamma oscillations and specifically PL of gamma oscillations to sound onset are enhanced when sounds are attended compared to passive listening, this effect being more pronounced in non-musicians than in musicians [46].

Musical sound stimuli are interesting from an evolutionary perspective as musical systems invented by humans are partly based on a generalized inherent preference for harmonic as opposed to rough frequency combinations [47–50]. In music, auditory unpleasantness or roughness in intervals or chords (i.e., combinations of two or more sounds, respectively) is referred to as sensory dissonance, whereas pleasant intervals or chords are termed consonant [51, 52]. Sensory dissonance occurs when the spectral sound components are unresolved leading to amplitude fluctuations or "beats" in the range 20–250 Hz [53, 54], whereas sensory consonance implies the absence of beats as well as a degree of harmonicity in the spectral components [55]. In Western tonal music, consonant chords, such as a major triad, are more commonly encountered, and hence prototypical, than less common or non-prototypical dissonant chords, such as an augmented triad or an altered seventh [56]. Interestingly, musicians rate dissonant chords as more unpleasant [57, 58] and have stronger autonomic nervous system responses to dissonance than non-musicians [59]. Moreover, in a previous MEG study [16], we found a stronger MMN to non-prototypical chords such as dissonant and mistuned ones, in musicians and comparable MMN responses to minor chords between musicians and non-musicians; the strength of the MMN to non-prototypical chords was also positively correlated with years of musical training. Overall these findings indicate that musicians, being more experienced with dissonance, might have a stronger ‘sound to meaning’ association and comply most stringently to music-theory expectations and affective connotations of dissonance when opposed to non-musicians.

Our main aim in this project was to investigate whether musical expertise modulates the strength of neuronal oscillations to musical chords and specifically whether this modulation would take place in oscillation amplitudes (AM) or oscillation phase dynamics (PL). Furthermore, we tested whether these changes in the oscillation dynamics would be specific to the non-prototypical chords, such as dissonant and mistuned ones, that are better known by musicians than non-musicians, hence reflecting experience-dependent modifications in neuronal processing networks. In contrast, the neuronal processing of major, and to a lesser extent also minor chords, that are familiar to any listener of Western tonal music (including those who never underwent formal musical training or played any instrument), should not be as strongly modulated by musical expertise. To this end, we recorded neuronal activity with MEG and examined the neuronal oscillatory dynamics by means of phase locking (PL) and amplitude modulation (AM) to common prototypical major and minor chords, and to less common non-prototypical dissonant and mistuned chords. The mistuned musical chord was included as an additional example of a non-conventional chord in Western tonal music, and functioned as a probe for the memory mechanisms that we aimed at studying. For instance, in individuals not acculturated to Western musical chords, a mistuned chord presented in isolation would not elicit a stronger response than a major or minor chord, since it does not contain any sensory roughness or inharmonicity in itself [16, 60]. In sum, based on the literature on ERs and brain oscillations to musical sounds, we hypothesized that both the phase locking and amplitude of gamma oscillations would be sensitive to experience-dependent plastic modulations of the neural responses to chords, and particularly to the salient non-conventional chords in musicians.

## Methods

### Participants

Twenty-six individuals with normal audiological status and no history of neurological disease participated in the study. Of these 13 are referred to as "musicians" and 13 as "non-musicians. Among these, the data from 2 musicians and 2 non-musicians were discarded from the analysis because of artifacts (large amplitude or frequent head motion, poor tuning of MEG system, excessive blinking), leaving 11 musicians and 11 non-musicians in the final analysis. The 11 musicians were all performing at a professional level and were either students from the Sibelius Academy of Music in Helsinki or educated musicians (3 males; mean age 25.2 ± 3.4; 1 left-handed; main instrument played: 4 piano, 2 voice, 1 clarinet, 1 cello, 1 flute, 1 organ, 1 violin; 18.2±4.5 years of musical practice; hours of daily practice 2±1). The non-musicians were individuals who had never played an instrument and had never received any formal music education (3 males; age 28.4 ± 4.2; 1 left-handed). The study was performed according to the Declaration of Helsinki with approval by the Ethical Committee of the Helsinki University Hospital". Participants signed a consent form, which had been reviewed and approved by the Ethical Committee. A subset of the participants of the present study was used in a previously published study focusing on the analysis of the MMN responses to the dissonant, mistuned and minor deviant chords [16].

### Stimuli

The stimuli were four types of musical triad chords recorded with a MIDI keyboard Roland A-33 connected to AKAI S-2000 sampler, and played with a realistically sounding piano timbre (AKAI sample library) (S1, S2, S3 and S4 Stimulus files). The chords were then edited using SoundForge and CoolEdit software to have the same intensity and duration (588 ms). In all the chords A4 was always the lowest (root) and E5 the highest note. To construct the four chords of the experiment ("major", "minor", "dissonant", and "mistuned"), the middle note was manipulated. The "major" chord was the traditional major triad, containing the major third interval between A4 and C#5. The "minor" chord was the traditional minor triad containing the minor third interval between A4 and C5. The dissonant chord contained several dissonant intervals, namely the minor second and the augmented fourth. This chord does not have a standard name in traditional music theory, due to its infrequent usage (however, see 52 for a post-tonal classification of chords). The "mistuned" chord had the middle note with a pitch between C and C sharp (C sharp decreased by 40 cents), thus sounding like a mistuned major chord. The frequency distance between the major chord and the mistuned chord was very small, having the middle pitch of the major chord, C sharp, a fundamental frequency of 554.4 Hz and the middle pitch of the mistuned chord, C sharp mistuned, a fundamental frequency of 541.9 Hz.

**Fig 1:**
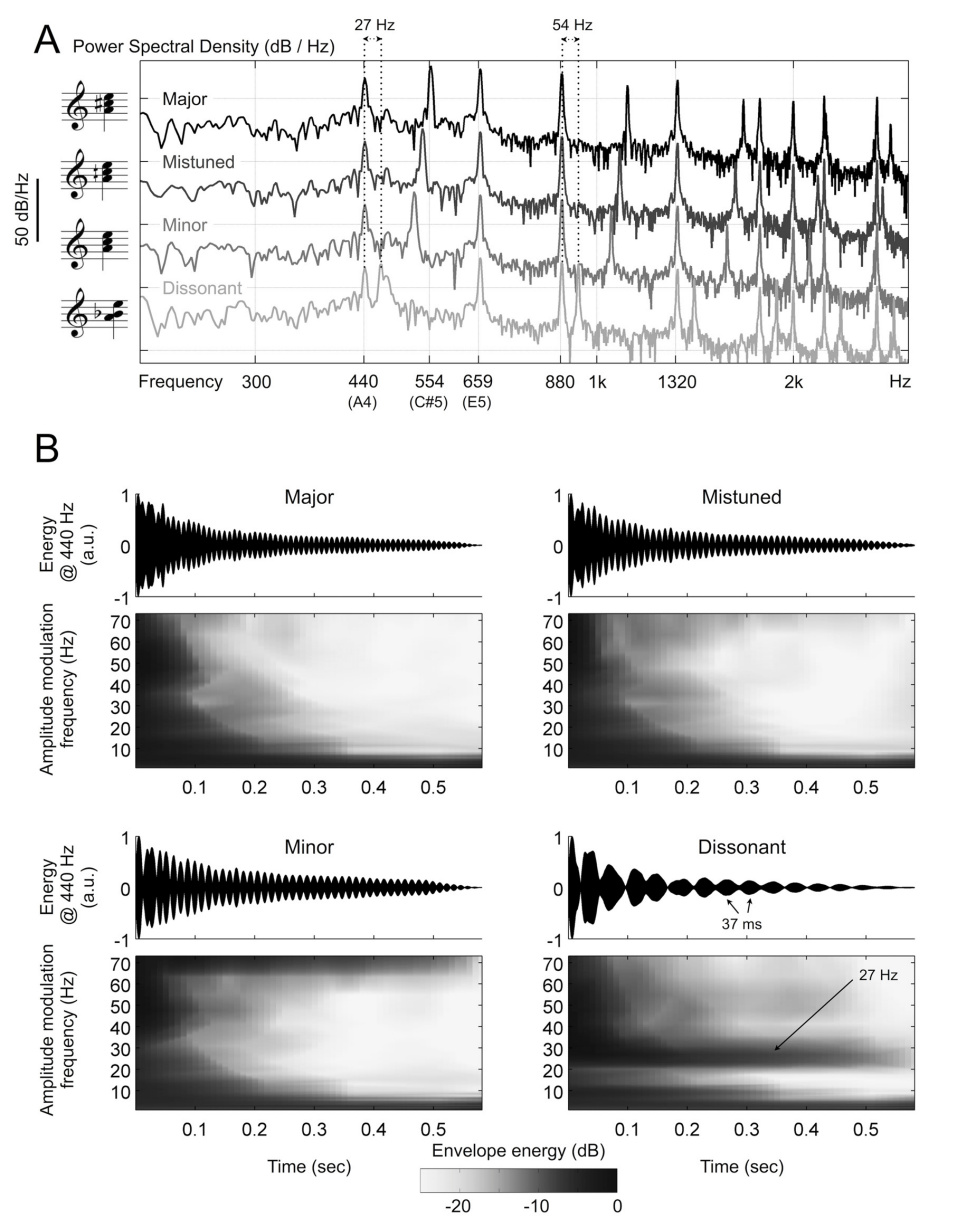
(A) Notation of the four stimuli used in the experiment. (B) Power spectral density plots (Welch's method) illustrate the formation of each chord by moving the middle tone in the 3-tone chords progressively further from C#5 (major triad). Harmonic overtones are illustrated up to 3 kHz. The frequency differences between the first and second tones of the dissonant chord (27 Hz), and their first harmonics (54 Hz), are indicated for the dissonant chord above the plot. (C) For each chord, the amplitude envelope of the signal in a narrow frequency band centered on the base tone (440 Hz) is plotted above the corresponding energy spectrogram (measured in dB, dark areas represent relatively higher energy than light areas). The dissonant chord is characterized by strong amplitude envelope modulation at a rate of 27 Hz, corresponding to a 37 ms period.

Analyses and illustrations related to the stimuli were implemented in Matlab (version 7.12, Mathworks, Inc.). Spectral density estimates were obtained for each chord using Welch's method (spectrum.welch, Hann window, 186 ms window length, 50% overlap) and are plotted in Fig 1A. The second tone of the dissonant chord is separated by approximately 27 Hz from the root tone (440 Hz). The difference between the first harmonics of the two tones is 54 Hz. We analyzed the temporal domain amplitude modulations for each chord in a narrow frequency band around the root tone by calculating the modulus of a Morlet wavelet centered on the root (for details on wavelet filtering, see below under "MEG data analysis"). The amplitude envelopes of each chord are plotted in Fig 1B (upper panels), along with their wavelet spectrograms (lower panels) in the frequency range used for MEG data analysis. As predicted by the spectral composition of the dissonant chord, the amplitude envelope around the 440 Hz tone is strongly modulated at approximately 27 Hz, corresponding to a period of *ca*. 37 ms. We repeated the temporal modulation analysis for the first harmonic of the root (880 Hz), the results of which are shown in Supporting Information (S1 Fig).

### MEG recordings

Neuronal activity was recorded in the BioMag Laboratory of the Helsinki University Central Hospital with a 306-channel VectorView whole-head MEG system (Elekta Neuromag, Finland). The signals from the 204 planar gradiometers were used in this study (on-line band-pass filter: 0.1–172.2 Hz; sampling rate: 600.615 Hz). Planar gradiometers are sensitive to local neuronal sources, whereby the spatial distribution of acquired field (gradient) patterns informs directly on the distribution of currents in the brain. The stimuli were presented at 60 dB SPL by the Presentation software (Neurobehavioral Systems Ltd.) through plastic tubes and silicon earpieces to subjects' ears. Before the experiment, the positions of four marker coils placed on the scalp were determined in relation to the nasion and both preauricular anatomical points with an Isotrak 3D-digitizer (Polhemus Inc., USA). The position of the magnetometer with respect to the head was determined for each experimental condition. Eye movements were monitored with vertical and horizontal bipolar electrooculograms (EOG). Trials with the electro-oculogram signal exceeding 150 μV were excluded from further analysis. The Maxfilter sofware (Elekta Neuromag Ltd., Finland) was used to suppress extra-cranial noise and to co-localize the recordings in signal space (SSS with temporal extension; correlation limit = 0.96, window length = 10 s, movement compensation to the "default" device coordinate origin [0,0,0] mm).

### Task

During the recordings, subjects were placed in supine position on a bed and were instructed to concentrate on watching a silent movie with subtitles projected onto the ceiling while ignoring the sounds. The chord stimuli were presented with a 1000 ms inter stimulus interval (ISI) in blocks of ca. 15 min duration. Each block included a chord presented frequently, the standard (p = .8), and a chord presented infrequently, the deviant (p = .2). The following combinations were presented: (1) major as standard and minor as deviant; (2) minor as standard and major as deviant; (3) major as standard and dissonant as deviant; (4) dissonant as standard and major as deviant; (5) major as standard and mistuned as deviant; (6) mistuned as standard and major as deviant. The presentation order of the conditions was pseudo-randomized across subjects. Hence, the stimulation paradigm was an ignore oddball type used for measuring the mismatch negativity (MMN), a change-specific neural evoked response to sounds [61]. MMN responses for the deviants sounds of the present study are published elsewhere [16]. Importantly, as randomization of the infrequent stimuli reduces habituation and fatigue in long recordings such as this, this paradigm is also very good for studying the role of neuronal oscillations under passive conditions. Hence, this paradigm allows the investigation of sensory encoding of musical sounds as well as automatic discrimination processes relying on feed-forward models. The experiment lasted approximately 3 hours including preparation, breaks and refreshments.

For the data-analyses, in order to maximize the amount of trials and to specifically avoid the strong transient responses associated with the deviants, we analyzed the responses to the standard chords. We extracted the standard chords that were preceded by at least 1 identical standard chord, and analyzed these non-attentively perceived stimuli. The accepted trials were balanced across the chord category (equal number of trials / chord) for within-subject analysis as PLF is a metric biased by the number of samples. The trials were selected iteratively from the conditions with more trials to match the condition with fewer trials. After artifact rejection and balancing, at least 200 trials per subject of each chord category were included in subsequent analyses.

### MEG data analysis

Each planar gradiometer channel, *X*(*t*), of the MEG time-series data was filtered into 34 frequency bands, *f*, *f* = 3…75 Hz, with a bank of Morlet wavelets *h*(*t*,*f*) [30] so that the complex filtered signal *Y*(*t*,*f*) is given by *Y*(*t*,*f*) = *X*(*t*) * *h*(*t*,*f*), where ‘*’ denotes convolution and *h*(*t*,*f*) = *A* e^(-*t* ^2^/2σ_t_^2^) e^(2*i*π*ft*). The time-domain standard deviation of the wavelet is given by σ_t_ = *m*/2π*f*, where the parameter *m* = 5 defines a compromise between time and frequency resolution, and *f* is the center frequency of the wavelet. The wavelet center frequencies were spaced one frequency-domain standard deviation, σ_f_ = *f*/*m*, from each other. We estimated the phase-locking (PL) of ongoing activity to the stimulus onset and the event-related oscillation amplitude modulation (AM) separately for each planar gradiometer. PL was quantified with the PL factor, PLF(*t*,*f*), that was given for each channel by PLF = *N* ^-1^ Σ_*n*_ (*Y*(*t*,*f*,*n*) / || Y(*t*,*f*,*n*) ||), where *n* = 1…*N* denote the trials and ||∙|| indicates the norm. PLV yields thus values between 0 and 1, where 0 indicates no phase-locking and 1 indicates full phase-locking. The averaged event-related amplitude envelopes, *A*(*t*,*f*), were given by *A* = *N* ^-1^ Σ_*n*_ || *Y*(*t*,*f*,*n*) || [30, 62]. PL and AM were estimated for each trial from 400 ms before, to 600 ms after stimulus onset. PL of ongoing activity to stimulus onset reflects the temporal precision at which ongoing oscillations time-lock to sensory stimuli, whereas AM reflects the strength of amplitude modulation.

Before the statistical group analysis, the individual subjects’ PLF(*t*,*f*) and A(*t*,*f*) were baseline corrected by subtracting the mean PL or AM values, respectively, calculated over the pre-stimulus period from −400 ms to −150 ms, separately for each wavelet central frequency. Subsequent statistical tests were performed on each time-frequency element (of PL and *AM*) and for each sensor. The baseline-corrected PL was tested against a null hypothesis of PLF = 0, by using the Wilcoxon-signed-rank test. Similarly, baseline-corrected amplitude values were compared to *A* = 0 using Student's t-test. To evaluate chord category and group effects on the oscillatory responses, we performed a 2-way ANOVA (chord: 4 levels, group: 2 levels) on both PLF and A values. The type 1 error rate was controlled throughout using a false discovery rate (FDR, [63]) corrected significance threshold of *p* < .01 over time and frequency samples. After FDR-correction with the threshold parameter q = 0.01, 1% of the elements for which the null hypothesis was rejected were expected to be false positives.

**Fig 2:**
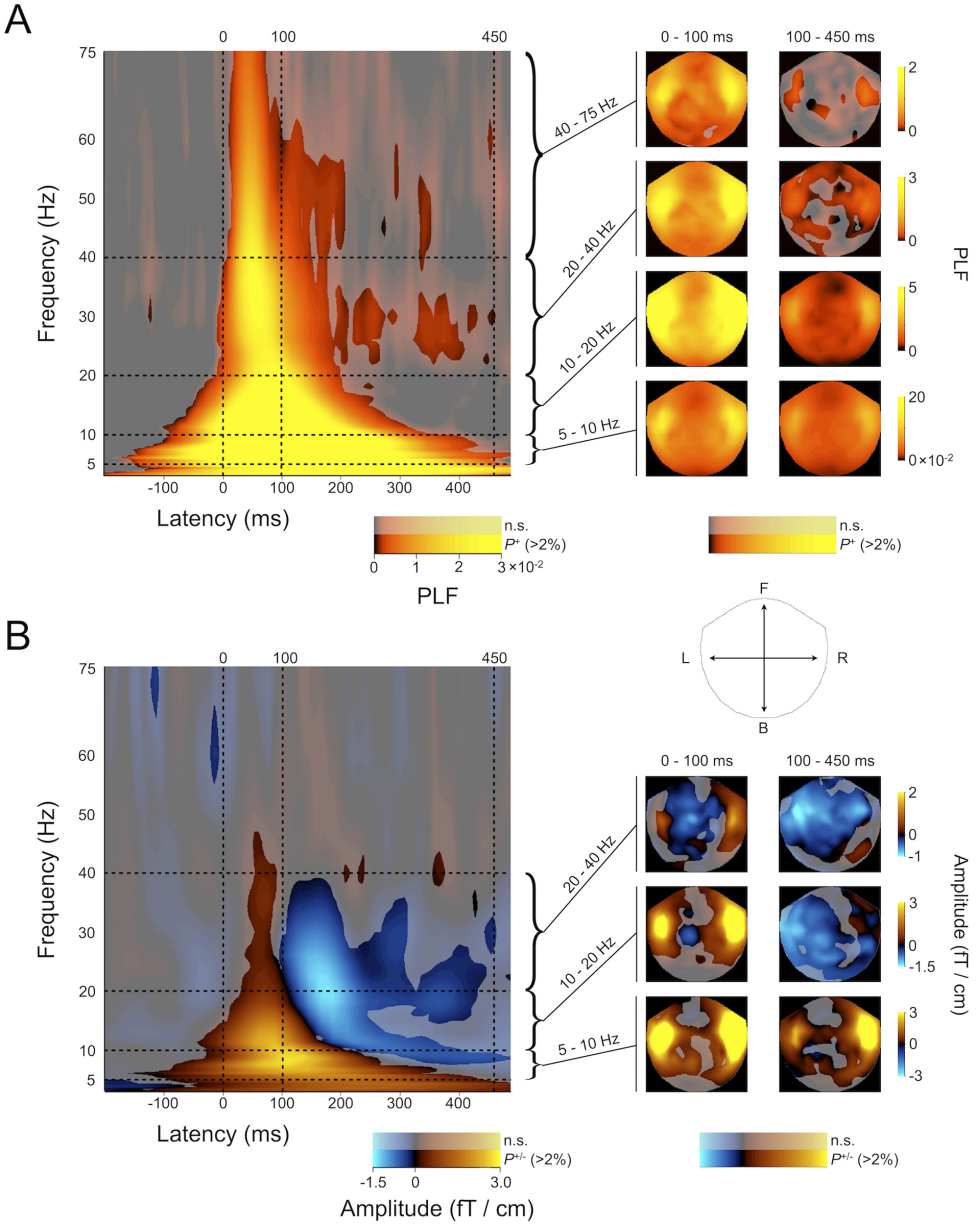
Time-frequency representations of MEG responses to musical chords reveal prominent stimulus-locking and amplitude modulation of oscillations in a wide frequency range (TFRs averaged over all subjects, chords, and over MEG planar gradiometers). A) *Left Panel*. TFR shows significant PL relative to the baseline period averaged over all subjects and all MEG-sensors (Wilcoxon's signed rank test, *p* < .01; FDR corrected). Robust PL between 3 and 75 Hz is observed both during early (0–100 ms) and late (100–400 ms) post-stimulus periods. Shaded areas represent TF-elements for which less than 2% of channels exhibited a significant effect. *Right panel*. The sensor topographies of selected TF-ROIs are illustrated in the sensor layouts. PL is strongest in the temporal channels. Shaded areas represent channels for which less than 2% of T-elements exhibited a significant effect. B) *Left Panel*. TFR shows significant AM relative to baseline averaged as in A (Student's t test, *p* < .01; FDR corrected). Positive AM is observed in a broad frequency range during the first few hundred ms. The positive AM is followed by negative AM in the alpha (8–12 Hz) and beta / gamma (18–40 Hz) frequency bands. *Right panel*. The positive AM is most pronounced in the bilateral temporal channels whereas the late negative AM in the 10 − 40 Hz range is observed in a slightly left-lateralized fronto-parietal and temporal network. Inset; Outline of the Elekta Neuromag Vectorview sensor helmet when flattened to 2D and seen from above (L = left, R = right, F = front, B = back).

To obtain comprehensive and unbiased visualizations of the results for a given test, we calculated time-frequency representations (TFRs) averaged over all sensors. In Figs 2 and 5B, the color scales relate to the average PLF and A values across the sensors. In all other TFRs, the color of each individual TF element represents the fraction (*P* ^+/-^, per cent) of sensors, in which a statistically significant positive and/or negative effect was observed for that particular TF element. We used a cutoff value *P+* > 2% throughout to filter out elements that were significant in only a few channels. To visualize the topographies associated with prominent effects observed in the TFRs, we selected time-frequency regions of interest (TF-ROIs). In Fig 2, the topographies show the average A and PLF values in each TF-ROI. In all other topographies, the color at each sensor position represents the fraction (*P* ^+/-^, per cent) of TF-ROI elements surviving the corrected statistical threshold. We emphasize that the topographies are for visualization purposes only, and that statistical conclusions should be based on the unbiased TFRs.

## Results

### Phase and amplitude dynamics associated with sound processing

We evaluated the modulations of oscillation phase and amplitude dynamics associated with the processing of musical chords. As described in Methods, PL reflects the temporal precision at which ongoing neuronal activity is time-locked to stimulus onset, whereas AM describes the mean peri-stimulus amplitude changes relative to the pre-stimulus baseline.

TFRs averaged across musicians and non-musicians as well as across the different chord types showed prominent PL across the whole analyzed frequency range (3–75 Hz) during the first few hundred milliseconds after stimulus onset (Fig 2A, Wilcoxon signed ranked test, *p <* .*01*, FDR corrected). Significant PL was observed for as long as 450 ms. The TFR of AM (Fig 2B) revealed an initial amplitude increase in the 3–40 Hz range, which was followed by amplitude suppression in a frequency range from 10 to 40 Hz (Fig 2B) (Student's t test, *p* < .01, FDR corrected).

The right panels in Fig 2A and 2B illustrate the sensor topographies of significant PL (A) and AM (B) of the MEG gradiometer signals with PLF and A values averaged across frequency bands and within early (0–100 ms) and late (100–400 ms) time windows. Both the PL and AM were modulated by the stimuli over wide areas across the scalp (illustrated by the insert in Fig 2). Strongest PL was consistently observed in the temporal channels, particularly so for responses in the beta and gamma frequency bands (20–75 Hz). The early positive AM showed a similar temporal topography being most pronounced in sensors over the temporal cortex. However, the late negative AM was observed in the channels over central, left fronto-parietal and left temporal cortices (Fig 2B, right).

### Phase and amplitude dynamics associated with musical expertise

To further evaluate whether the observed PL and AM effects were associated to musical expertise or inherent to the chord stimulus type, we subjected the PL and AM data to separate 2-way ANOVAs with factors Group and Chord.

**Fig 3:**
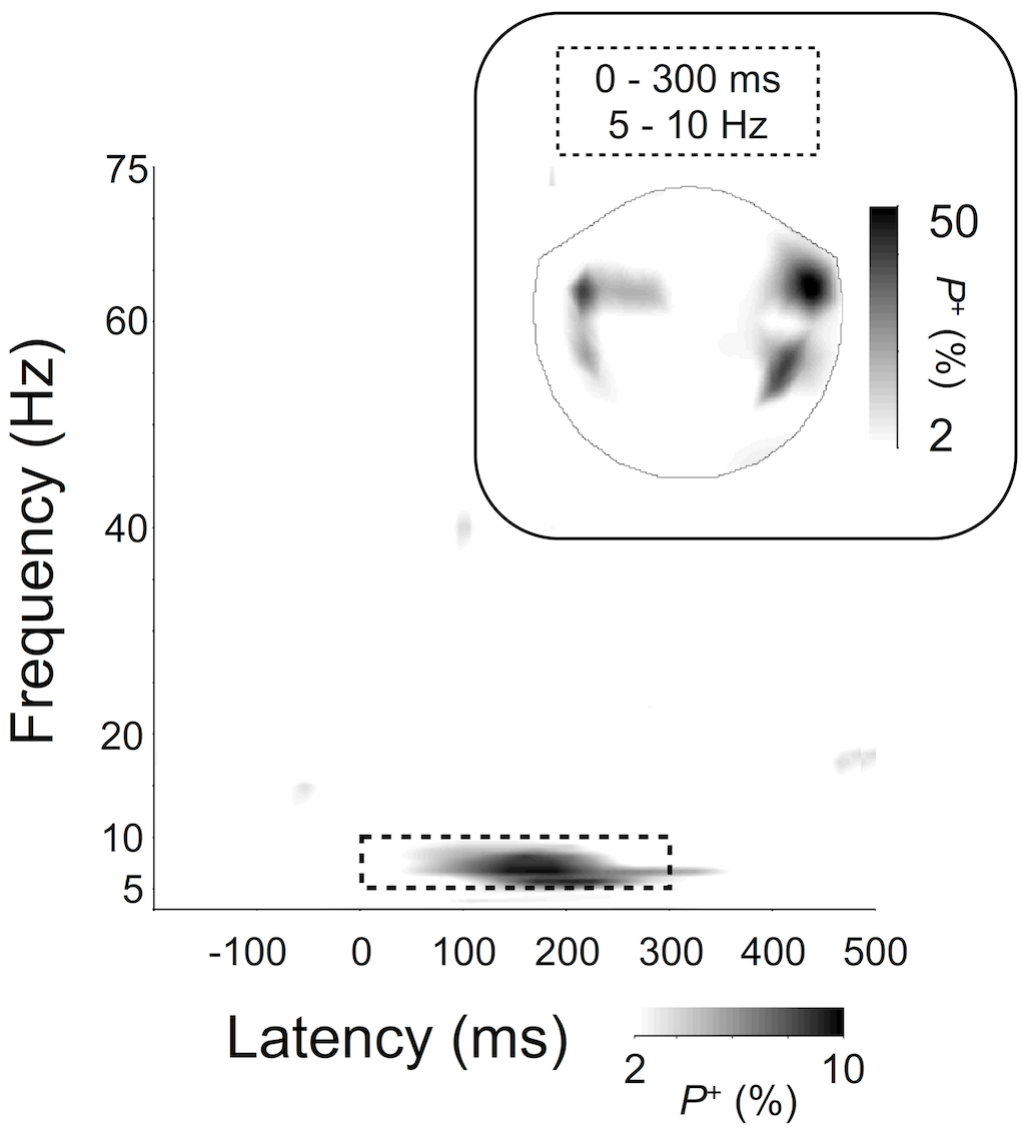
Main effect of musical expertise (Group) on response amplitude (2-way ANOVA, *p* < .01; FDR corrected) induced by musical chords. A group difference in AM was observed 5 to 10 Hz range from 0 to 300 ms post-stimulus, and localized to bilateral temporal sensors (insert). Only those TF-elements (channels) that survive the test of *p* < .01 (FDR-corrected) in at least 2% of channels (TF-elements) are shown (see Methods for details).

For stimulus-induced AM, we found a main effect of Group only between 5–10 Hz and during the first 0–300 ms (Fig 3, ANOVA, *p* < .01, FDR-corrected), with larger AM in musicians compared with non-musicians, particularly in bilateral temporal sensors (right column). No evidence for Chord-by-Group interactions (p> 0.05) was observed.

**Fig 4:**
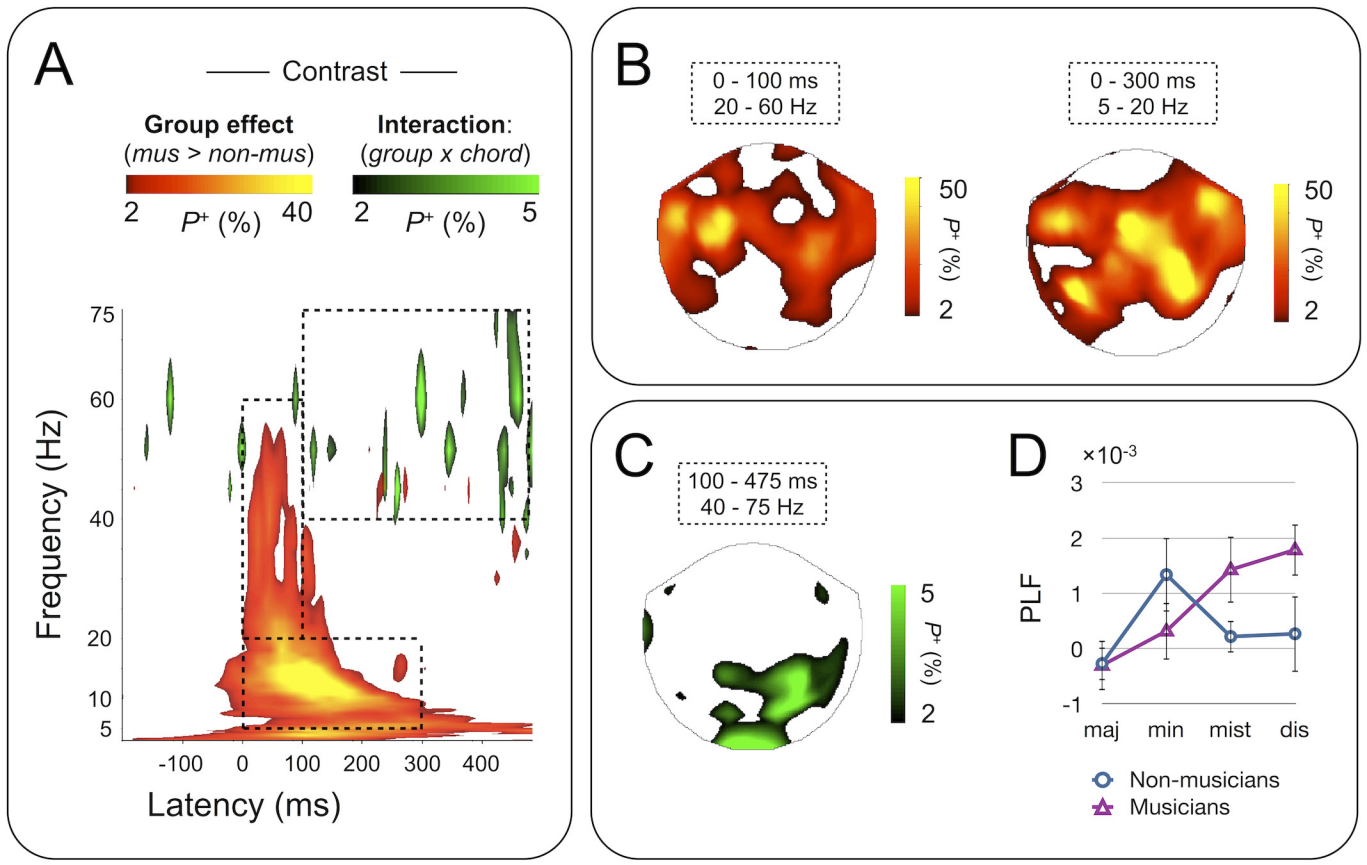
Main effect of musical expertise (Group) and Group by Chord interaction on PL (2-way ANOVA, *p* < .01; FDR-corrected) induced by musical chords. A) A main effect of Group (musicians > non-musicians, red-yellow color scale) was observed in a wide frequency range between 3 and 60 Hz, and a Group by Chord interaction (black-green color scale) in the gamma band. Only those TF-elements that survived the test of p < .01 (FDR-corrected) in at least 2% of channels are shown. B) The sensor topographies for the selected TF-ROIs show the spatial distribution of the main effect of Group, where only those channels that survived the test of p < .01 (FDR-corrected) in at least 2% of TF-elements are shown. Musicians exhibited stronger PL in a widespread network, with pronounced effects in temporal, parietal and central sensors. C) The sensor topography for the significant Group by Chord interaction (green regions in panel A) reveals a cluster of right-hemispheric channels over the parietal cortices. D) Post-hoc analysis of the Group by Chord interaction. The channel subset shown in C was used to extract the PLF values (within-group mean +/- SD) for each chord category.

For PL data, we also found a main effect of Group, with musicians exhibiting stronger early (0–100 ms) PL than non-musicians at frequencies between 3 and 60 Hz (Fig 4A, *p* < .01, FDR-corrected; for sensor topographies see Fig 4B). Furthermore, we observed a Chord-by-Group interaction in the gamma (40–75 Hz) frequency band during the late post-stimulus period (100–475 ms), localized to the right-hemispheric posterior channels (Fig 4C).

To gain insight into this finding, we extracted, for each subject and chord category, the PL values in the time-frequency range and channel subset shown in Fig 4C. As circular analyses yield to inflated statistical observations [64], the purpose of this post-hoc analysis is merely to visualize the contribution of each chord type PL to the observed group-by-chord interaction effect. The mean (+/- SD) PL values for the four levels of the chord category-factor are plotted in Fig 4D, separately for musicians and non-musicians. We thus found that in both groups, PL to major chords was the weakest, and the relative strengths of PL to minor, mistuned and dissonant chords differed between groups. Specifically, in non-musicians PL to minor dominated over the non-prototypical and major chords, whereas the situation was reversed in musicians. In Musicians, PL to non-prototypical chords was stronger than that to major or minor chords.

### Phase and amplitude dynamics associated with chord type

**Fig 5:**
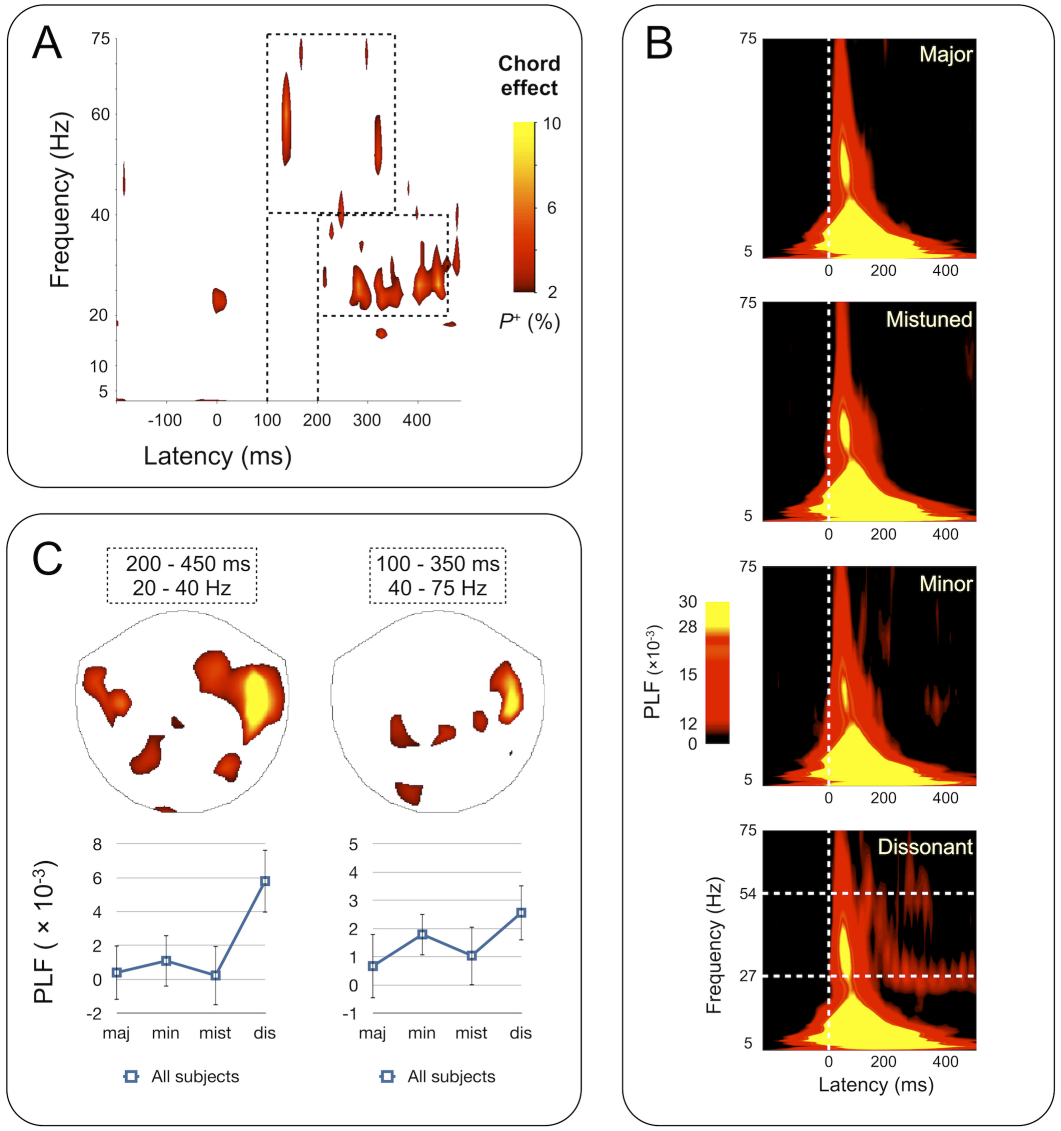
Main effect of Chord on PL (2-way ANOVA, p < .01; FDR-corrected) induced by musical chords. A) Significant main effect of Chord is observed in the 20–40 Hz and 40–75 Hz bands, between 100 and 450 ms after stimulus onset (TFR thresholded as in Fig 4). B) TFRs of PL, separately for each chord type, averaged over subjects. Note that the color scale is intentionally chosen to emphasize mid-range PL values (red). C, above). The sensor topographies (above) in selected TF-ROIs (dashed boxes in A) for which at least 2% of TF-elements exhibited significant effects (p < .01; FDR-corrected). The main effect of Chord was observed predominantly in the right-hemispheric sensors in both ROIs. C, below). Post-hoc analysis of the PL values (mean over subjects +/- SD) in the TF-regions in A and sensor selection in C (above) indicates that the observed effect is caused by PL of population oscillations to the periodic amplitude envelope fluctuation of the dissonant chord (see also Fig 1).

No main effects of chord types were found for oscillation amplitudes (data not shown). By contrast, during the late post-stimulus period PL exhibited a significant main effect of Chord (2-way ANOVA, *p* < .01, FDR-corrected) prominently in the 20–40 Hz and also in the 40–75 Hz frequency bands (Fig 5A). Fig 5B shows the TFRs of the mean PLF values associated with each chord and reveals strong PL at 27 Hz for the dissonant chord type, which corresponds to amplitude envelope modulation of the sound stimulus (cf. S1 Fig). The 54 Hz modulation of the 880 Hz component is also highlighted (cf. S1 Fig). To reveal the sensors showing the main effect of chord type, we averaged the PLF values across beta / low-gamma (20–40 Hz) and middle / high-gamma (40–75 Hz) bands. The sensor topographies revealed that for two TF-ROIs the main effect of Chord was observed in the right hemispheric temporal sensors (Fig 5C). To further characterize PL in these sensors, we performed a post-hoc analysis, in which we extracted for each subject and chord category the mean of PLF values across the TF-ROI and sensor selection. The mean (+/- SD) PLFs for the four levels of the chord category-factor (Fig 5C) showed that the main effect of Chord was driven by PL to the dissonant chords so that PL to these chords was stronger than PL to the other chords.

## Discussion

We investigated using MEG recordings whether phase-locking of ongoing oscillations to stimulus onset (PL) and oscillation amplitude modulation (AM) to musical chords vary between different chord types and between musicians and non-musicians. The strength of PL varied as a function of both chord type, and musical training, whereas the strength of oscillation AM depended only on musical expertise. AM to chords was increased in musicians only in the low frequency range (5–10 Hz), but the PL was enhanced over a broad frequency range in both experimental groups (3–75 Hz). A post-hoc analysis of the Group by Chord interaction revealed an effect in right-parietal sensors (100–475 ms), in the form of enhanced PL to dissonant chords in musicians and to minor chords in non-musicians. We also found that the beat frequency in dissonant chords, *i*.*e*., the physical amplitude modulation of the sound stimulus, was detectable in the MEG signal across subjects, irrespectively of their musical expertise.

### Musical expertise is reflected in enhanced PL in response to musical chords

Our aim was to characterize the implications of musical expertise with respect to phase and amplitude dynamics of brain oscillations triggered by three-tone chords played with a realistically sounding piano timbre. In Fig 4A we show that in musicians, the early PL to the presentation of musical chords is enhanced compared to non-musicians in the sensors over the parietal and temporal cortices over a wide frequency range from theta to gamma frequency bands during the first ~300 ms, *i*.*e*., during the traditional time-averaged ER component. Oscillation amplitudes were also stronger in musicians compared to non-musicians, but only in a limited (5–10 Hz) frequency band and channel subset (see Fig 3). We thus conclude that the temporal precision of event-related dynamics is specifically enhanced by musical expertise, rather than overall strength of the response.

As the strengthened PL in musicians compared to non-musicians took place in the time-window of the ERs, our results suggest that common observations in the literature of the effects of musical training on the ERs, including our previous study on a subset of the data presented here [16], could be partially due to enhanced PL of ongoing oscillations. Our PL findings are in line with the data showing that phase resetting of ongoing alpha oscillations contributes to the generation of the auditory ERs [65, 66]. In other words, the neural mechanism underlying the superior psychoacoustic acuity and neural representation of salient sounds in musicians might derive from a more synchronized firing of cortical neurons in response to sound features, rather than or in addition to a larger spatial recruitment of neuronal populations. The latter prevalent view on the neural mechanisms governing music-derived plasticity was supported by evidence of enlarged gray matter of the Heschl’s gyrus and the planum temporale in musicians [67, 68]. Our findings do not contradict the anatomical observations but provide additional knowledge on the neurophysiological correlates of musical expertise. Furthermore, the effect of musical expertise on the phase rather than oscillation amplitude dynamics is consistent with the findings, which show that training in the auditory domain modulates the phase of the theta-band oscillations [69]. Oscillation phase-dependent coding is indeed central in the neural processing of auditory stimuli. Phase-dependent coding is evident in association with behavioral performance of auditory tasks [70], but also in the delta and theta band phase-entrainment, such as during the discrimation of speech stimuli [71], and attending a stream of audiovisual stimuli [72–74]. Our results therefore add to a growing body of literature on the importance of phase based coding in the auditory system.

### Gamma-band PL reflects chord specificity and musical expertise

PL in the gamma- but not in the lower-frequency bands was sensitive to the chord-type. These results support and extend prior data [26, 27] in that phase-locked gamma oscillations signal the encoding of auditory stimulus features. Taken together, phase-locked gamma oscillations have been observed only for language and music therefore supporting the view that auditory evoked gamma oscillations may reflect the memory match or feed-forward template for well-learned auditory stimuli [26, 42, 43].

Here we found a significant Group by Chord interaction in the long-latency gamma band PL (Fig 4A) in posterior parietal sensors (Fig 4C) that was specifically due to a difference in the relative strength of PL to minor, mistuned and dissonant chords. In detail, PL to minor chords was stronger than to other chords in non-musicians, whereas in musicians PL to the non-prototypical mistuned was stronger than to the other chords. Hence, these results are in line with our hypothesis and show that in addition to behavioral and automatic neural responses observed for the mistuned non-prototypical chords in musicians [16], also the neuronal phase dynamics to these sounds is modulated by musical experience.

Further, in contrast to minor, mistuned, and dissonant chords, PL to the major chord, which forms the basis of most Western music styles and is thus highly familiar to most people, was weak in both groups, suggesting that its processing was achieved with relatively weak effort therefore supporting the idea of existing feed-forward templates for these well-learned stimuli. Repetition of sensory stimuli leads to both increases and decreases of neuronal activities depending on the number of repetition. Previous studies which have reported both decreased [75, 76] and increased [77] gamma band response amplitudes for learned versus novel stimuli therefore show that the relationship between the strength of neuronal responses and learning is not straightforward. Indeed experience dependent modification in sensory processing networks are complex involving both the modification in the feed-forward processing networks as well as dynamics of recurrent interactions [42]. Thereby, taken that gamma band PL reflects the representation of musical chords, musical experience may both increase and decrease the strength of neuronal gamma oscillations depending on the phase of learning.

Musicians are daily exposed to a wide variety of sounds, and our musician participants were mainly classical instrumentalists, listening to the classical music style, which contains a larger variety of chords than those present in the mainstream pop-rock genre mainly preferred by non-musicians.

Taken together, the results suggest that the enhanced gamma-band PL in musicians compared to non-musicians for the non-prototypical chords may reflect enhanced familiarity with these sounds encountered less often by non-musicians. In contrast, the increased PL to minor chords in non-musicians compared to that of mistuned and dissonant chords, possibly reflect the moderate familiarity of minor chords to non-musicians, these chords being commonly used in Western popular music, in contrast to the non-prototypical chords that are and less frequently heard by non-musicians in their daily music consumption.

These data suggest that familiarity of sound stimuli derived from the individual listening and playing biography may account for some of the observed variability. For instance, musical training has been shown to strengthen evoked as well as induced gamma band responses in musicians but only to the timbre of the played instrument [45]. We speculate that elevated gamma band amplitude and/or PL may reflect increased allocation of processing resources towards moderately familiar stimuli, which are subjectively salient but yet not well learned. The purpose of such an allocation might be to modify existing feed-forward templates to include features of the unfamiliar sounds or reflect the matching of moderately unfamiliar sensory stimuli with existing templates.

### PL responses to the amplitude envelope of musical chords

In contrast to group-by-chord interactions observed in the high-gamma band, the long-latency beta- / low-gamma band PL component showed a main effect of chord irrespective of the musical expertise. This effect corresponded to the main peaks of the sound amplitude envelope of the dissonant chords, namely the 27 Hz modulation of the root 440 Hz tone (A4) and 54 Hz modulation of the first harmonic of the root (880 Hz). This result suggest that PL to the amplitude modulation of a complex sound previously observed in intracranial measurements of responses to tone intervals [78] is also detectable in MEG measurements to sounds of ecologically valid duration and loudness profile. Previous studies showed that the discernment of pitch of dissonant sound spectra that contain poorly resolved frequency components is supported by neuronal PL to the temporal periodicity of the amplitude-modulated sound envelope in cell populations in the primary auditory cortex [79–83]. Perception of sensory dissonance in tone intervals correlates with the strength of PL responses of primary auditory cortex ensembles to the amplitude envelope "beats", measured intracranially [78]. The propensity of neuronal populations in the human auditory cortex to phase-lock its activity to stimulus amplitude modulations can also be observed extracranially in EEG and MEG measurements in the auditory steady-state response (ASSR), which are obtained by adding (artificial) periodic amplitude/frequency modulations to the stimuli [84–87]. In contrast to previous observations showing that repeated sound exposure advances the phase of the ASSR both in attended and non-attended conditions [88], we did not find evidence that musical expertise modifies the degree of long-latency PL to the amplitude envelope of dissonant chords in either hemisphere. Hence, we suggest that our results identify the auditory cortical PL response to the amplitude-modulated envelope as a central neural mechanism for dissonance encoding that is relatively resistant to plastic remodeling. However, we did not apply a direct test of the neural envelope following response. Such a test would involve localising an auditory source and cross-correlating its signal to the stimulus envelope (e.g. [89]). Further studies specifically optimized to study this proposition are warranted.

The observed PL to the stimulus amplitude envelope was stronger in the right hemisphere, in accordance with the hypothesis that the relative distribution of cells that synchronize (temporal encoding) or do not synchronize (rate encoding) to repetitive acoustic events may differ between the hemispheres [90]. More generally, the “asymmetric sampling in time” (AST) hypothesis, predicts that processes that require analysis of extended samples of the input signal (100–300 ms duration) are lateralized to the right hemisphere, compared with the shorter time-scale processes lateralized to the left (20–50 ms) [91]. Results from both human psychophysical [92] and monkey electrophysiological [79] experiments indicate that the encoding of pitch of unresolved harmonics requires analysis of waveform periodicity and, as a consequence, longer temporal integration windows (on the order of 100 ms) than when signal components can be resolved spectrally (~25 ms) [93, 94].

## Conclusions

In the present study, we show that musical expertise is reflected in generally enhanced PL of ongoing oscillations to the onset of musical chords. Long-latency gamma band responses were sensitive to chord type and hence likely reflected sound feature representation. Musicians had strengthened PL to non-prototypical chords while non-musicians had strengthened PL to minor chords. Hence, the neuronal coupling to chords exhibited a complex relationship with musical expertise and stimulus type, which may reflect formation and modification of neuronal sound representations. We also observed right-lateralized PL corresponding to the amplitude envelope of dissonant chords, but did not find evidence for its modulation by musical expertise, suggesting that it could be part of a relatively “hard-wired” processing system routing the sampling of temporally slower events to the right hemisphere.

In sum, our findings of synchronized phase coupling to dissonant frequency content in all subjects and of enhanced PL to salient non-prototypical chords in musicians well comply with the latest view on (musical) expertise. Diverging from the initial proposal by Ericsson and colleagues [95], which emphasizes the role of deliberate practice over any innate ability, researchers currently agree with viewing expertise as an integrated composite of innate dispositional traits, cognitive abilities and deliberate practice, having a strong genetic component [96, 97].

We also observed right-lateralized PL corresponding to the amplitude envelope of dissonant chords, but did not find evidence for its modulation by musical expertise, suggesting that it could be part of a relatively “hard-wired” processing system routing the sampling of temporally slower events to the right hemisphere. Further studies on auditory learning-induced plasticity that specifically control for familiarity and salience factors are warranted in order to disentangle the effects of the temporal sharpening of neuronal responses to sound stimuli, reflected as increased PL, from amplitude changes.

## Supporting Information

S1 Fig “Amplitude envelope modulation of the sound stimuli”. (TIF)

S1 Stimulus File “Major chord”. (WAV)

S2 Stimulus File “Minor chord”. (WAV)

S3 Stimulus File “Dissonant chord”. (WAV)

S4 Stimulus File “Mistuned chord”. (WAV)
