# Supplementary figures and images for: Experience Drives Synchronization: The phase and Amplitude Dynamics of Neural Oscillations to Musical Chords Are Differentially Modulated by Musical Expertise
Source: PLoS One. 2015 Aug 20;10(8):e0134211. doi: 10.1371/journal.pone.0134211 (PMC4546391; doi:10.1371/journal.pone.0134211)

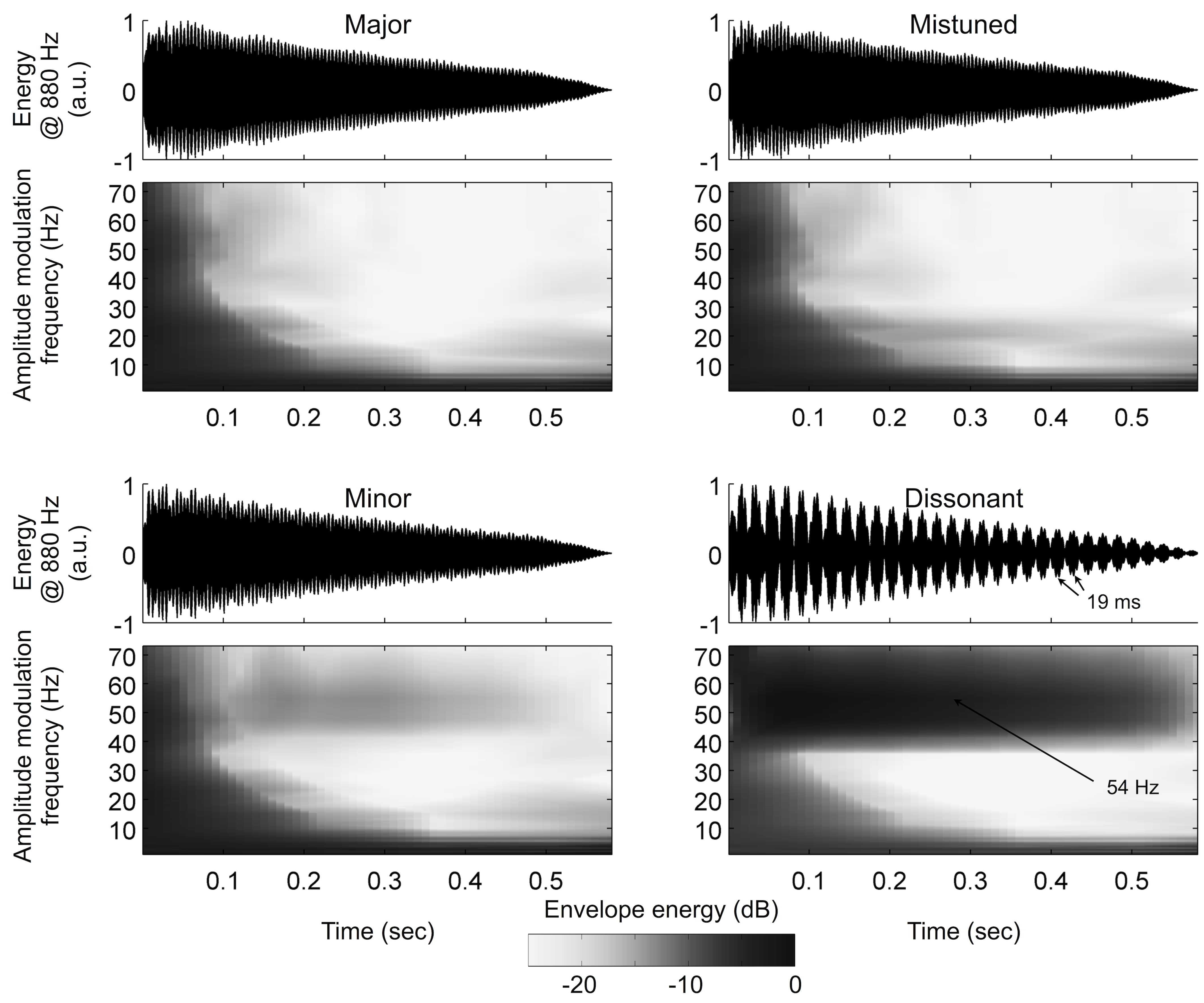

Supplement: S1 Fig — (TIF) [file pone.0134211.s001.tif]
